# Pancreatic abscess within hepato-gastric ligament: case report of an extremely rare disease

**DOI:** 10.1186/s12893-020-0688-0

**Published:** 2020-01-30

**Authors:** Sabyasachi Bakshi

**Affiliations:** 1 Department of General surgery, BSMCH, Bankura, West Bengal PIN-722102 India; 2 Kathghara Lane, Sonatuli, Hooghly, West Bengal PIN-712103 India

**Keywords:** Pancreatic abscess, Infected pancreatic pseudocyst, Hepato-gastric ligament, Extra-pancreatic pseudocyst

## Abstract

**Background:**

Pancreatic pseudocyst is a very common benign cystic lesion of the pancreas. It develops in 5–15% of patients with peri-pancreatic fluid collection following acute pancreatitis. Collection usually occurs within the lesser sac of the omentum (near the pancreatic head and body region). But in 20–22% cases, that may be extra-pancreatic like in the mediastinum, pleura, in the peritoneal cavity including the pelvis. The pancreatic pseudocyst typically contains brownish fluid with necrotic tissue sludge which may get infected giving rise to infected pseudocyst or pancreatic abscess. The present case is an unusual condition of a young alcoholic subject who was finally diagnosed as a case of a pancreatic abscess within hepato-gastric ligament and was managed with operative intervention.

To the best of the author’s knowledge, it is the first-ever reported case of a pancreatic abscess within the hepato-gastric ligament in the world. Literature was reviewed to explore potential etiopathogenesis and therapeutic strategies of this extremely rare condition.

**Case presentation:**

A 38 years old gentleman, chronic alcoholic, having a previous history of acute pancreatitis 3 months back, presented with fever (102 degrees Fahrenheit) and a huge [20 cm (horizontal) X 15 cm (vertical)] severely painful swelling in the epigastric region. The swelling was round-shaped, intra-abdominal, fixed to deeper tissue, tense-cystic, poorly trans-illuminant, non-pulsatile and irreducible. Routine blood tests showed leucocytosis (14,500/mm^3^) with neutrophilia and elevated plasma pancreatic amylase and lipase levels. USG and MDCT scan of the whole abdomen revealed a thick-walled echogenic cystic swelling of size 18 cm × 12 cm in the epigastric region. USG guided aspiration of the cyst revealed mixed purulent brownish fluid. The cyst fluid was negative for mucin stain and contained high amylase level with low CEA level, suggesting infected pancreatic pseudocyst.

An open drainage procedure was considered through an upper midline laparotomy**.** Aspiration of the pus mixed cyst fluid along with tissue debris was done. Through irrigation of the cyst was done with normal saline. The cyst wall was de-roofed leaving a small part adherent to the inferior surface of the left lobe of the liver. Later the cyst fluid culture showed significant growth of *Escherichia coli*. He was put on IV antibiotics. The patient was discharged in a stable condition after 5 days. The histopathological examination confirmed pancreatic abscess.

Six months after the operation, the patient is doing well, remaining asymptomatic and there is no sign of recurrence.

**Conclusions:**

Due to extreme rarity, pancreatic abscess formation within hepato-gastric ligament may be a diagnostic dilemma and requires a high index of suspicion. Surgeons should be aware of this rare clinical entity for prompt management of potential morbidity.

## Background

The pancreatic pseudocyst is a very common benign cystic lesion of the pancreas. It develops in 5–15% of patients with the peri-pancreatic fluid collection, usually 4 weeks after the initial attack of acute pancreatitis. Although collection usually occurs within the lesser sac of the omentum (near the pancreatic head and body region), in 20–22% cases, that may be extra-pancreatic [1] like in the mediastinum, pleura, and anywhere in the peritoneal cavity including the pelvis [1, 2]. Pancreatic pseudocysts are commoner in cases with alcohol-induced acute pancreatitis (20%) compared to gallstone induced one (6.6%) [3]. Intra-hepatic extension of pancreatic pseudocyst may be seen rarely when the sequestered fluid extends through the hepato-duodenal or hepato-gastric ligaments. [4] In extreme rarity (very few reported cases) pancreatic pseudocyst may be found located within the hepato-gastric ligament. The pancreatic pseudocyst typically contains brownish fluid mixed with necrotic tissue sludge which may also sometimes get infected giving rise to an infected pseudocyst or pancreatic abscess. The present case is an unusual condition of a young alcoholic subject who presented with huge epigastric painful cystic mass and fever. He was finally diagnosed as a case of a pancreatic abscess within hepato-gastric ligament and was managed with operative intervention.

To the best of the author’s knowledge, it is the first-ever reported case of a pancreatic abscess within the hepato-gastric ligament in the world. Due to extreme rarity, pancreatic abscess formation within hepato-gastric ligament may be a diagnostic dilemma and requires a high index of suspicion. Literature was reviewed to explore potential etiopathogenesis and therapeutic strategies of this extremely rare condition.

## Case presentation

**Fig. 1:**
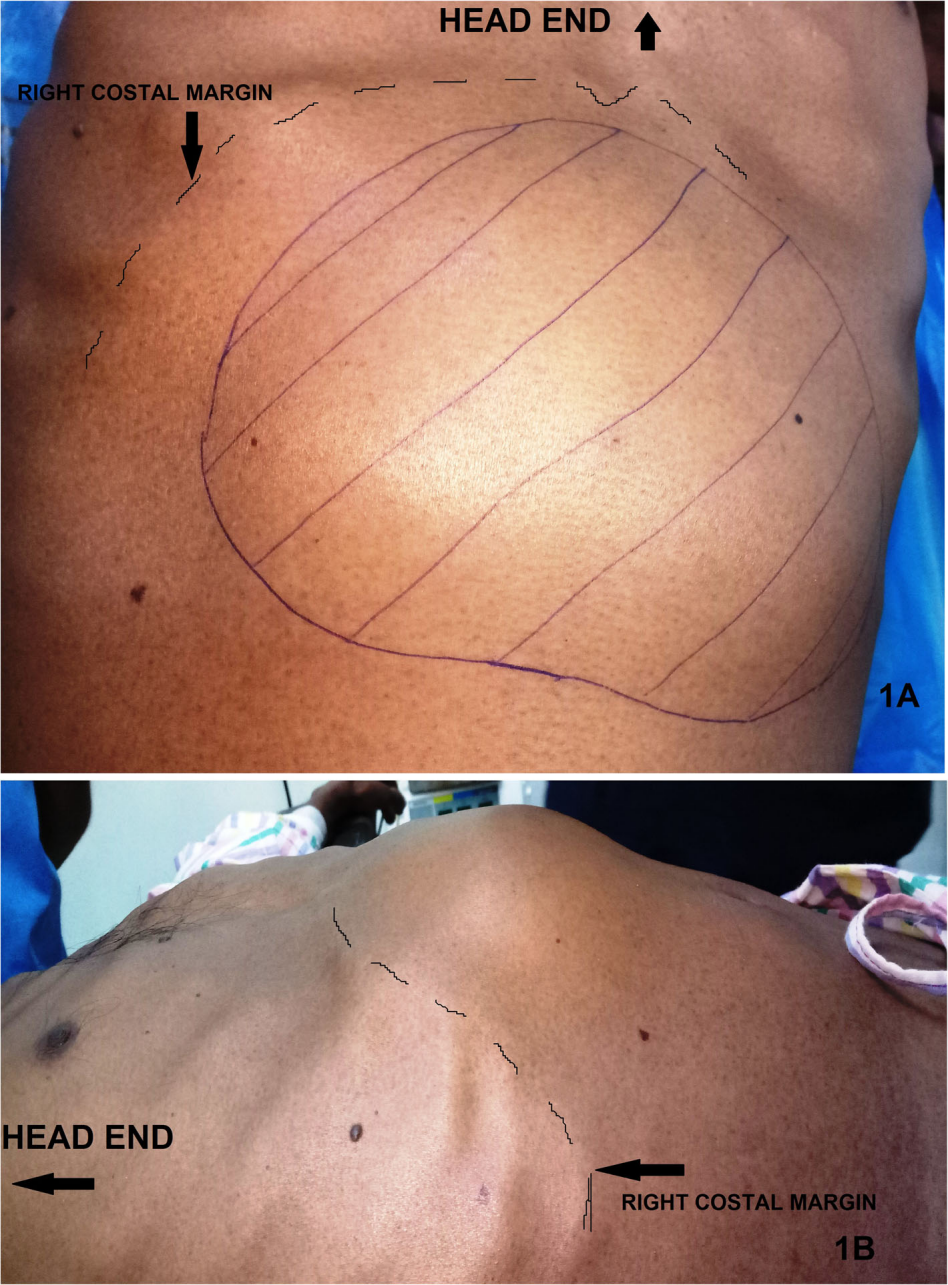
Preoperative images of the huge epigastric swelling (1A = Anterior and 1B = Right Lateral view)

A 38 years old gentleman, chronic alcoholic, having a previous history of a single episode of hospitalization with acute pancreatitis 3 months back, presented with fever (102 degrees Fahrenheit) and a huge severely painful swelling in the epigastric region **(**Fig. 1**)**. The swelling, initially mild dull aching and smaller in size, started 2 months back and gradually attained the present size [20 cm (horizontal) X 15 cm (vertical)] in the last 7 days. He gradually developed tenderness and induration over the swelling after a trivial blunt trauma on the area 7 days back. General examination revealed a normotensive person (BMI of 19.6) without any ascites, dysuria or any sign of intestinal obstruction. He was significant only for the swelling which was located in the epigastric region. The swelling was round-shaped, intra-abdominal, fixed to deeper tissue, tense-cystic, poorly trans-illuminant, non-pulsatile and irreducible. The swelling showed no expansile impulse on coughing and was devoid of any bowel sound within it. He neither had a history of any previous abdominal surgery nor had he received any treatment for this disease. He had no history of coagulopathy or similar illness in his family.

**Fig. 2:**
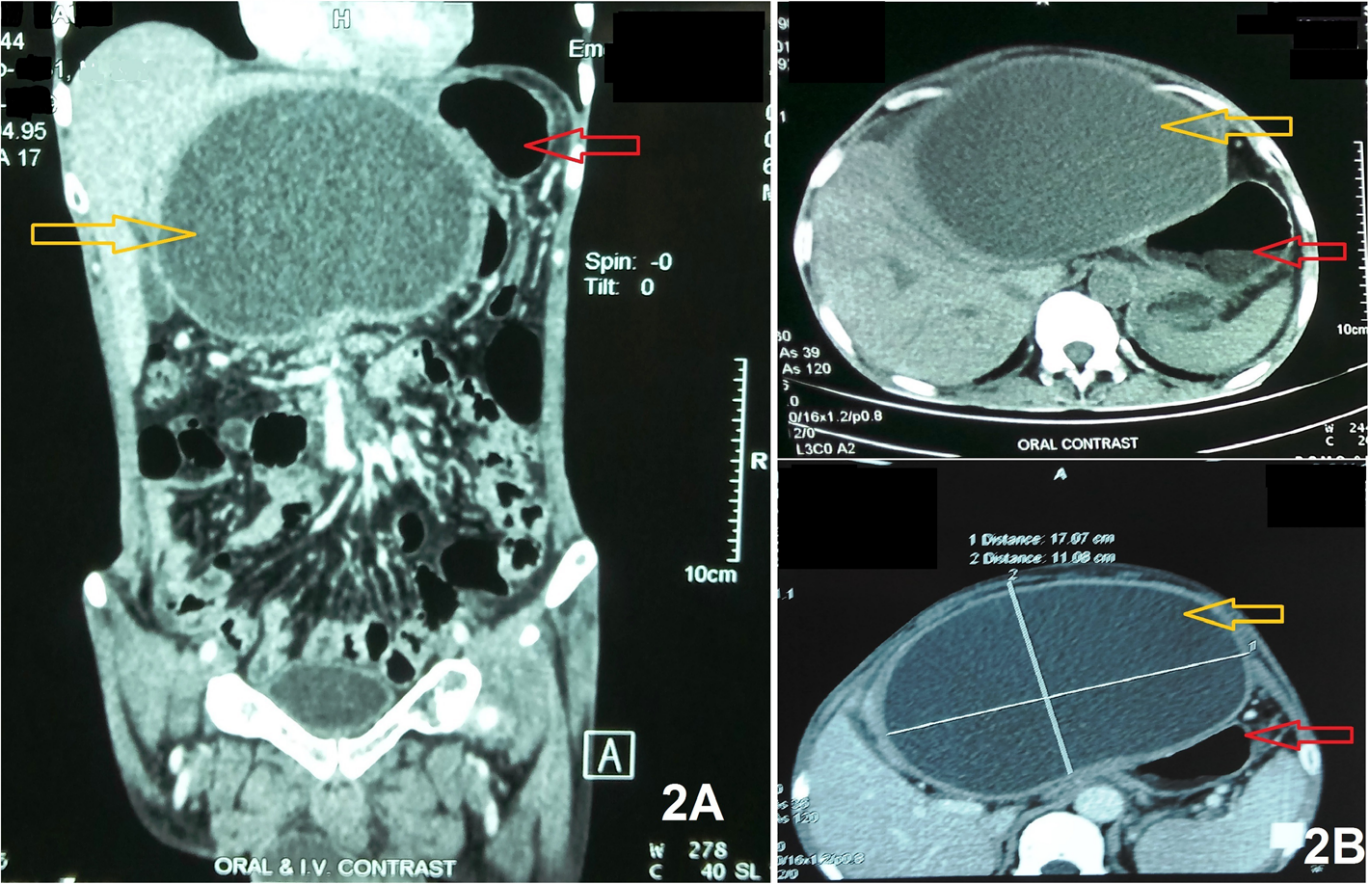
Pre-operative whole abdominal MDCT Scan images.2A = Coronal view, 2B = Transverse/cross-sectional view. Yellow arrows show solitary pancreatic abscess (echogenic exophytic) located between the left lobe of the liver and anterior surface (Red arrows) of the stomach without communication with any organ. 2B clearly shows that the cyst is separated from liver tissue

The possibility of pyogenic liver abscess, infected hydatid cyst, post-traumatic infected hematoma or infected pseudocyst of the pancreas was considered clinically. Urgent relevant blood tests and urine analysis were within normal limits except leucocytosis (14,500/mm^3^;reference range 4000-8000/mm^3^) with neutrophilia (81%) and elevated plasma pancreatic amylase(342 IU/L;reference 20-96 IU/L) and lipase (456 IU/L;reference 3-43IU/L) levels. Ultrasonography (USG) of the whole abdomen revealed a thick-walled echogenic cystic swelling of size 18 cm × 12 cm in the epigastric region, anterior to the stomach and inferior to the left lobe of the liver without any communication to adjacent organs and there were no ascites. Colour Doppler flow study failed to show any vascular component of this swelling. USG guided needle aspiration of the cyst revealed mixed purulent brownish fluid. Cytological examination of the fluid showed inflammatory cells but no malignant cell was found. The fluid was sent for bacterial culture and antibiotic sensitivity tests. The cyst fluid examination was negative for mucin stain and contained high amylase level (8680 IU/L) with low CEA level. Cyst fluid study was suggestive of infected pancreatic pseudocyst. Contrast-enhanced Multi-Detector CT Scan with the pancreatic protocol (MDCT) of the abdomen revealed a large (17.07 cm X 11.08 cm) well defined enhancing thick-walled (6 mm) exophytic cystic space-occupying lesion (SOL) that was located inferior to the left lobe of the liver, displacing the stomach posteriorly **(**Fig. 2**).** MRCP failed to show any communication of the cystic SOL with pancreatic ducts.

**Fig. 3:**
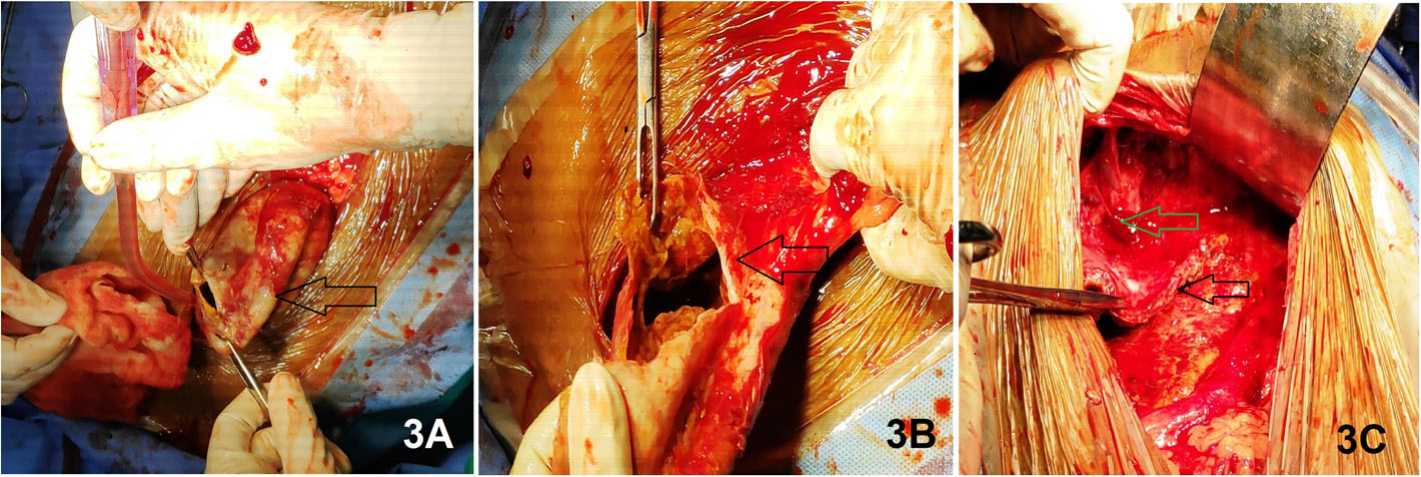
Intra-operative finding of a thick-walled (image 3A) cyst (Black arrows) containing purulent fluid and tissue debris (image 3B). Most of the cyst wall is free from surrounding tissues except (shown in image 3C) small portion over the inferior surface of the left liver lobe (Green arrow)

**Fig. 4:**
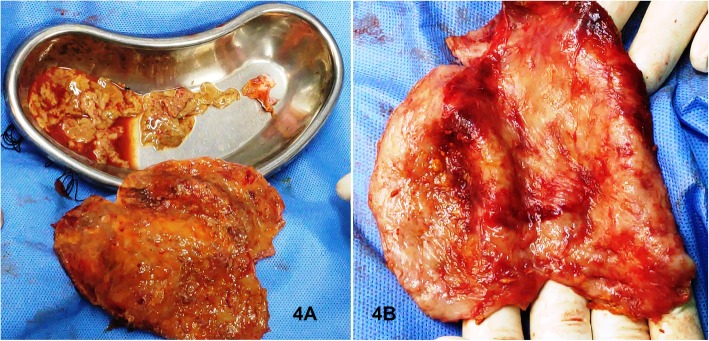
Excised thick fibro-collagenous cyst wall with tissue debris after thorough wash.4A = Inner surface,4B=Outer surface

**Fig. 5:**
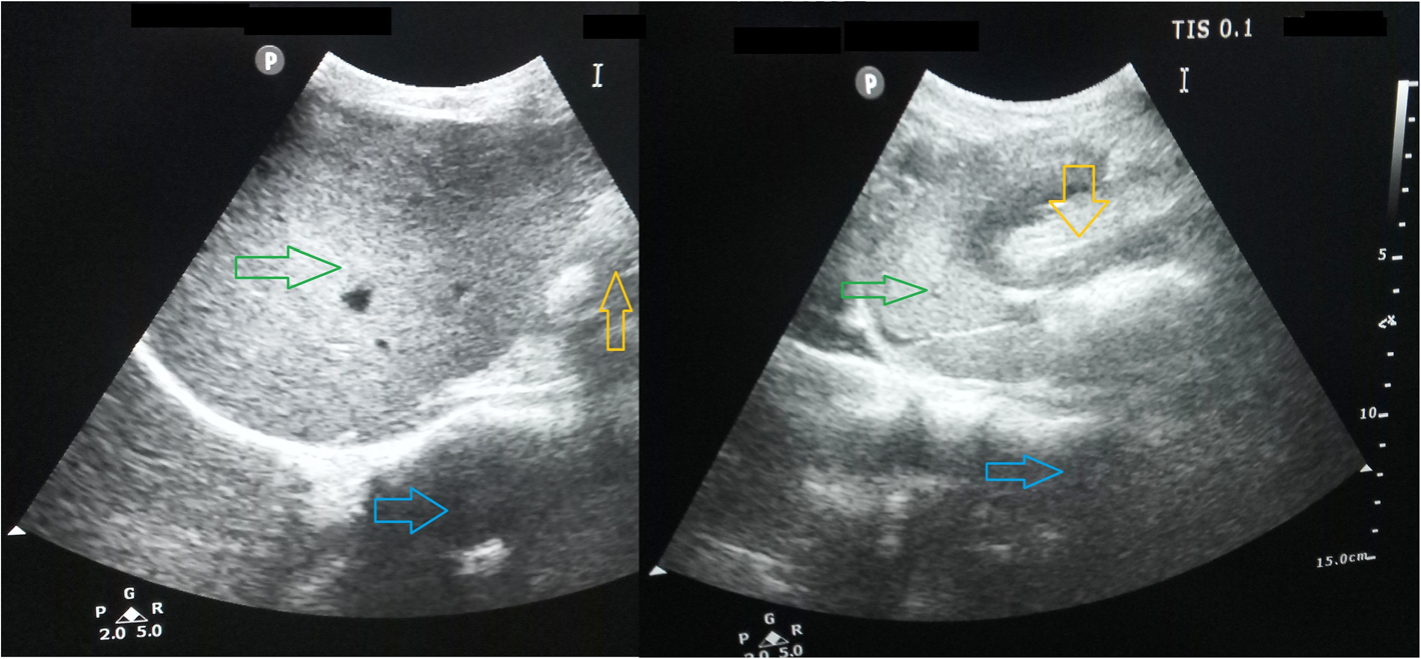
Post-operative Day 3 greyscale Ultrasonography of the liver (Green arrows) and epigastric region revealed the position of hypo-echoic omental fat (Yellow arrows) that was placed below the left lobe of the liver during operation and position of the pancreas (Blue arrows).USG shows no demonstrable collection in the operative field

After proper resuscitation and pre-operative workup of the subject, considering the presence of (on pre-operative USG Scan) high volume non-drainable tissue debris and abscess formation within the cyst, open drainage procedure was considered through an upper midline laparotomy. On entering the abdomen, one large thick-walled cyst was identified within the hepato-gastric ligament. **(**Fig. 3**).** Aspiration of the thick pus mixed cyst fluid, along with tissue debris, was done avoiding any spillage in the abdomen. Through irrigation of the cyst was done with normal saline. The cyst wall was de-roofed leaving a small part that was adherent to the inferior surface of the left lobe of the liver. **(**Fig. 4**)** A portion of omentum was securely placed over the cleaned inner surface of the residual cyst wall. **(**Fig. 5**)** Abdomen was closed leaving a positive pressure closed abdominal drain over the operative site. The cyst wall and tissue debris were sent for histopathological examination.

His postoperative stay in the hospital was uneventful. The cyst fluid culture showed significant growth in *Escherichia coli*. He was put on IV antibiotics according to the drug sensitivity report. Oral feeding was started the next day. He became afebrile after 2 days and early ambulation was encouraged. On the third postoperative day, after removal of the drain, total collected 110 ml of serosanguinous fluid from the abdomen was again sent for study. Post-operative serum amylase, lipase level came to be normal as well as pancreatic enzymes level in drain fluid was also found negligible. There was no surgical site infection. The patient was discharged in a stable condition after 5 days.

**Fig. 6:**
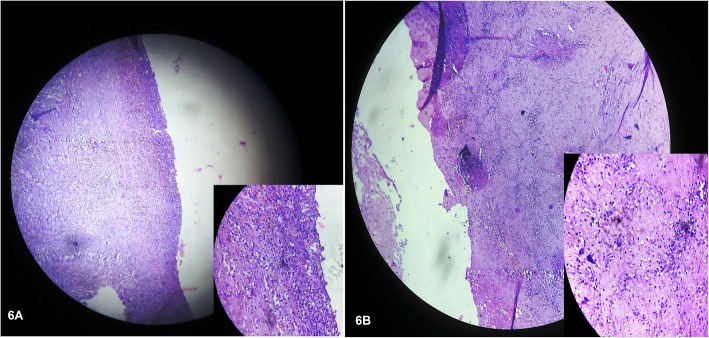
**a** Sections from the cyst wall revealed compressed fibro-collagenous tissue (but no epithelium) infiltrated with mixed inflammatory cells (rich in neutrophils, macrophages, lymphocytes, and plasma cells). (Enlarged image in Inset). **b** Sections from debris tissues showed features of necrosis with inflammatory infiltrates without any granuloma or malignancy (Inset image), suggesting chronic infective cystic lesion

The histopathological confirmation of pancreatic abscess was made when multiple sections from the cyst wall showed features of compressed fibro-collagenous tissue infiltrated by mixed inflammatory cells (rich in neutrophils, macrophage, lymphocytes, and plasma cells). The tissue debris also showed features of necrosis with rich inflammatory infiltrates **(**Fig. 6a & b**)** suggesting chronic infective cystic lesion. Although no feature of granuloma or malignancy could be detected.

Six months after the operation, the subject is asymptomatic and has returned to his usual life without any clinical sign of recurrence. He was advised regular follow up in our surgical Out-Patient Department (OPD) to detect any recurrence by clinical examination and ultrasonography.

## Discussion and conclusions

Pseudocyst of the pancreas is a very common cystic lesion of the pancreas which comprises 75% of all cysts of the pancreas. The other 25% are cystadenomas, IPMNs, cystic necrosis of adenocarcinoma, and congenital polycystic disease. Pancreatic pseudocyst develops in 5–15% of patients with peri-pancreatic fluid collection following acute pancreatitis. It occurs when sequestered pancreatic fluid collection localizes and fibrotic reaction completes usually 4 weeks after the initial attack of acute pancreatitis, in or around the pancreas. But 20–40% of the pseudocyst of the pancreas resolves spontaneously. Trauma and recurrent/chronic pancreatitis may cause rupture of pancreatic ducts which also gives rise to pseudocyst formation due to increased ductal pressure. The capsule of the pseudocyst is composed of collagen and granulation tissue with a lining fibrin layer which lacks epithelium [5].

Although collection usually occurs within the lesser sac of the omentum, it can also be found in another ectopic (extra-pancreatic) sites depending on where the pancreatic enzymes were released and the path it had traveled. Most frequently it is localized within the pancreatic head and body region, but in 20–22% cases, they may be extra-pancreatic [1] in a location like in the mediastinum, pleura, and anywhere in the peritoneal cavity (commonly spleen, stomach wall) including the pelvis [1, 2]. Pancreatic pseudocysts are more commonly found in alcohol-induced acute pancreatitis (20%) than with gallstone induced one (6.6%) [3]. Intra-hepatic pancreatic pseudocyst formation may be found rarely when the sequestered fluid extends through hepato-duodenal or hepato-gastric ligaments. [4]

Several proposed mechanisms explain how pseudocyst of the pancreas occurs after acute pancreatitis in different extra-pancreatic locations. These are as follows-.

1. Up to 8 th weeks of intra-uterine life, ventral mesentery contains ventral anlage of the pancreas and the remnant of the dorsal portion of the ventral mesentery remains after birth as gastro-hepatic and gastro-duodenal ligaments. So, pancreatic sequestered fluids from the head of the pancreas after acute pancreatitis can spread towards porta hepatis, left lobe of the liver [4, 6] or even into the right lobe of the liver [7] along with the attachment of hepato-gastric ligament

Hepato-gastric and Hepato-duodenal ligaments are peritoneal folds that are continuous with lesser omentum. The Hepato-gastric ligament contains the coronary vein and left gastric artery while it connects the liver with lesser curvature of the stomach. And the Hepato-duodenal ligament attaches liver with the duodenum while transmitting portal vein, hepatic artery, common hepatic duct and part of the cystic duct through it.

2. In extreme rarity (very few reported cases) pancreatic pseudocyst may be found located within the hepato-gastric ligament when ascend of pancreatic fluids get arrested within it. The pancreatic pseudocyst typically contains brownish fluid with necrotic tissue sludge. These materials may get infected commonly by gram-negative rods (*Escherichia coli*) giving rise to infected pseudocyst or pancreatic abscess.
3. Pancreatic fluids may leak into pre-renal space after the rupture of the main pancreatic duct or its branches. This may also cause pseudocyst formation later.
4. The posterior layer of peritoneum may get eroded and pancreatic fluid can accumulates in the lesser sac and can follow the hepato-gastric ligament.
5. Uncommonly, in the event of pancreatic duct rupture posteriorly into the retroperitoneum, the fluid may go into the mediastinum [8]. The pancreatic fluid may also enter the mediastinum via natural hiatuses (esophageal, vena caval or aortic), foramen of Morgagni and rarely by direct penetration of the diaphragm [9].

Clinically presented with palpable epigastric mass and rarely hepatomegaly (if the liver lobe is involved) [10], up to 50% of subjects with pseudocysts of the pancreas develop symptoms including persistent pain or recurrent pain after the initial resolution, early satiety, nausea, and weight loss. In the case of infection and abscess formation, there may be toxic manifestations like pain, pyrexia, hypotension, tachycardia, leucocytosis. Differential diagnosis of pseudocyst of the pancreas in hepato-gastric ligament includes tubercular abscess, left liver lobe abscess, hematoma, teratoma and necrotic tumor of the hepato-gastric ligament. Teratoma (fatty, round, sharp margin with calcification on MDCT) and congenital cysts (mesenteric, duplication, omental or choledochal cyst) may be present in the hepatoduodenal ligament. [11]

The clinical diagnosis is corroborated by MDCT or MRCP which is very helpful especially in the case of extra-pancreatic locations. MRCP, in addition to information given by MDCT like cyst-wall thickness and nature of contained fluid (hemorrhagic/infective), can show communication with other organs. The presence of gas bubbles may be seen in the case of abscess formation on radiological assessment. Endoscopic ultrasound (EUS) with FNAC may be needed in whom the diagnosis is unclear, as rarely pseudocyst may form due to underlying malignancy. Characteristics features of pancreatic pseudocyst aspirate include high amylase level but low CEA level and absent mucin. Serum amylase level, ten times greater than normal, has 98% specificity and 73% sensitivity for detecting pseudocyst of pancreas. [12]

Observation is indicated for asymptomatic patients with cyst diameter lesser than 4 cm, located in the pancreatic tail region without any duct obstruction/communication as they may undergo spontaneous resolution [5]. Pseudocysts, with greater than 10 mm wall thickness, multiple, persistent for more than 6 weeks with associated pancreatic duct pathology and increasing in size on follow-up, are unlikely to resolve spontaneously and require intervention [13].

Invasive therapies are indicated for symptomatic patients and when the possibility of a cystic neoplasm can’t be ruled out. When most of the pancreatic pseudocyst, if needed, is treated with decompressive procedures rather than resection, pancreatic abscess in ectopic sites like within hepato-gastric ligament should be resected/de-roofed after proper drainage (avoiding spillage) and thorough irrigation.

There is increasing evidence that transduodenal/transgastric endoscopic drainage of non-infected thick-walled pseudocyst is safe and effective modality when it is located in close contact (< 1 cm) with duodenum or stomach respectively [14]. There should not be any pseudoaneurysm, portal hypertension, and gastric varices if the endoscopic procedure is tried. In this process endoscopically diathermic puncture is made on the wall of duodenum or stomach over the cyst and a drainage system is introduced. Inadvertent arterial bleed and infection is the complication of this process. Alternatively, transpapillary drainage can be attempted when communication with the main pancreatic duct is patent. Endoscopic dilatation and stent placement may be tried when duct stricture is encountered [15].

Surgical (open/laparoscopic) intervention is needed for failed or unsuitable endoscopic cases. Cystogastrostomy or cystoduodenostomy should be done, as internal drainage procedures, where the cyst is located near the stomach or duodenum respectively. The cysts, which are not in contact with those two organs, need Roux-en –Y cystojejunostomy.

Surgical cysto-enterostomy is highly successful in achieving immediate drainage in more than 90% cases with 12% chances of recurrence in the long term depending upon location and etiology. Although this procedure carries 1–5% mortality and 10–30% morbidity [13].

Percutaneous external drainage has a high incidence of external fistula formation, so it may be tried in septic (high-risk group) patients secondary to pseudocyst infection/abscess only. Percutaneous drainage may also be tried in case of an unruptured, noninfected cyst with the intact pancreatic duct. While simple aspiration is associated with a 70% recurrence rate, placement of percutaneous drainage catheter provides a 90% recurrence-free resolution [16]. Another advocated method of treatment may be catheter drainage (introduced through the hepato-gastric ligament, between stomach and spleen, or trans-gastric route) combined with octreotide (somatostatin hormone analog). Octreotide decreases both the basal and the stimulated pancreatic enzymes secretion [17]. Catheter drainage is contraindicated [13] if cyst fluid is very thick and mixed with non-drainable necrotic tissues, in the presence of arterial pseudo-aneurysm and active hemorrhage. It is also contraindicated in case of suspected malignancy and lack of safe entry site.

To the best of the author’s knowledge, it is the first-ever reported case of a pancreatic abscess within the hepato-gastric ligament in the world.

Despite the extreme rarity, pancreatic abscess formation within hepato-gastric ligament may be a diagnostic dilemma and requires a high index of suspicion. Patients with a pancreatic abscess in hepato-gastric ligament may present with toxic features and epigastric lump. After proper resuscitation and confirmation of the disease, the cyst should be resected/de-roofed after proper drainage (avoiding spillage) and thorough irrigation. Proper antibiotics, according to culture and sensitivity test of cyst fluid, should be administered. Surgeons should be aware of this clinical entity during the management of suspected pathology involving the epigastric region for prompt management of potential morbidity.

## Data Availability

Presented within the manuscript, please contact the author for additional data requests.

## Abbreviations

BMI: Body Mass Index
CEA: Carcino Embryonic Antigen
EUS: Endoscopic Ultrasonography
FNAC: Fine Needle Aspiration Cytology
IPMN: Intraductal Papillary Mucinous Neoplasm
IV: Intra-venous
MRCP: Magnetic Resonance Cholangio-Pancreatography
USG: Ultrasonography
